# Supplementary material for: A mosaic-type trimeric RBD-based COVID-19 vaccine candidate induces potent neutralization against Omicron and other SARS-CoV-2 variants
Source: eLife. 2022 Aug 25;11:e78633. doi: 10.7554/eLife.78633 (PMC9481243; doi:10.7554/eLife.78633)
Supplement: Figure 1—source data 1. [file elife-78633-fig1-data1.zip › Figure 1 - source data 1/SDS-PAGE analysis results.pdf]

ImageQuant TL

Analysis Report: 20211224-4 .tif

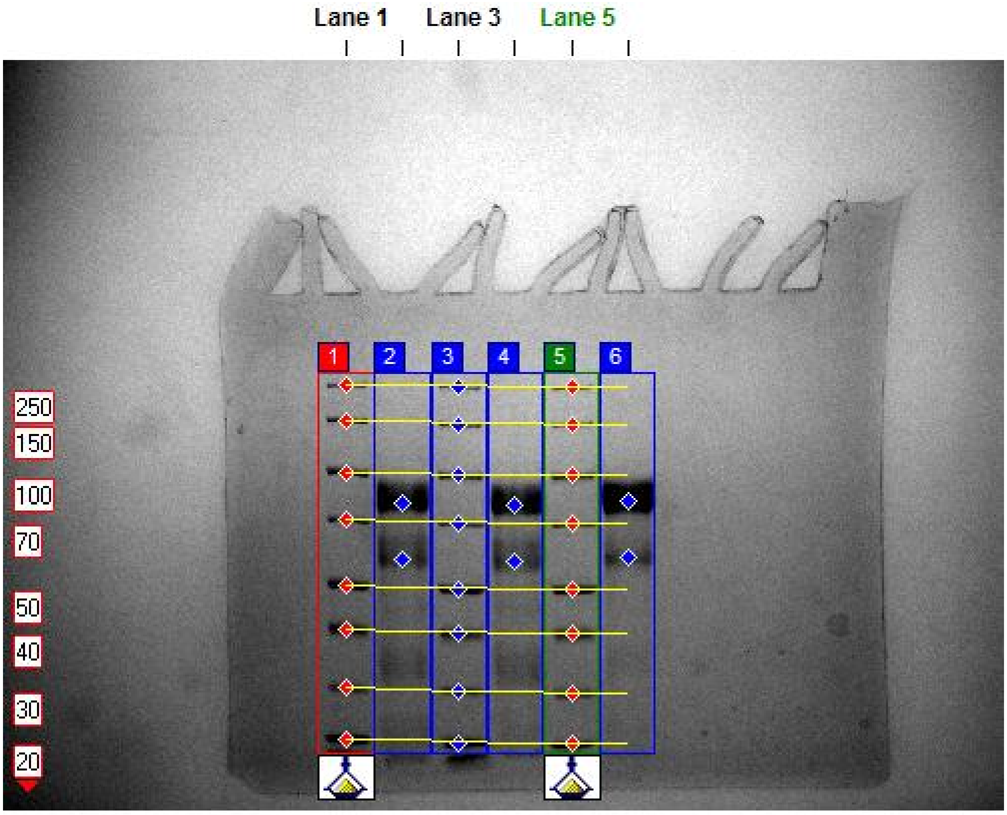

Lane Table

Lane	Bands	Band Volume	Lane Volume
Lane 1	8	48556442.55	53952444.72
Lane 2	2	51042462.03	64020396.86
Lane 3	8	48534114.72	53387088.97
Lane 4	2	55569960.34	68873771.44
Lane 5	8	44157964.42	48880860.59
Lane 6	2	69755415.23	77450159.42

Lane Data Report: 20211224-4 .tif, Lane 1

Summary

**Name:**	Lane 1

Background

**Background Type:**	Rolling Ball
**Rolling Ball Radius:**	200

Band Detection

**Automatic Detection:**	Yes
**Minimum Band Slope:**	50
**Median Filter:**	10
**Percentage Maximum Peak:**	0
**Maximum Peak Measure:**	Gel
**Edge Detection Method:**	Automatic Edges
**Band Positions Edited:**	No

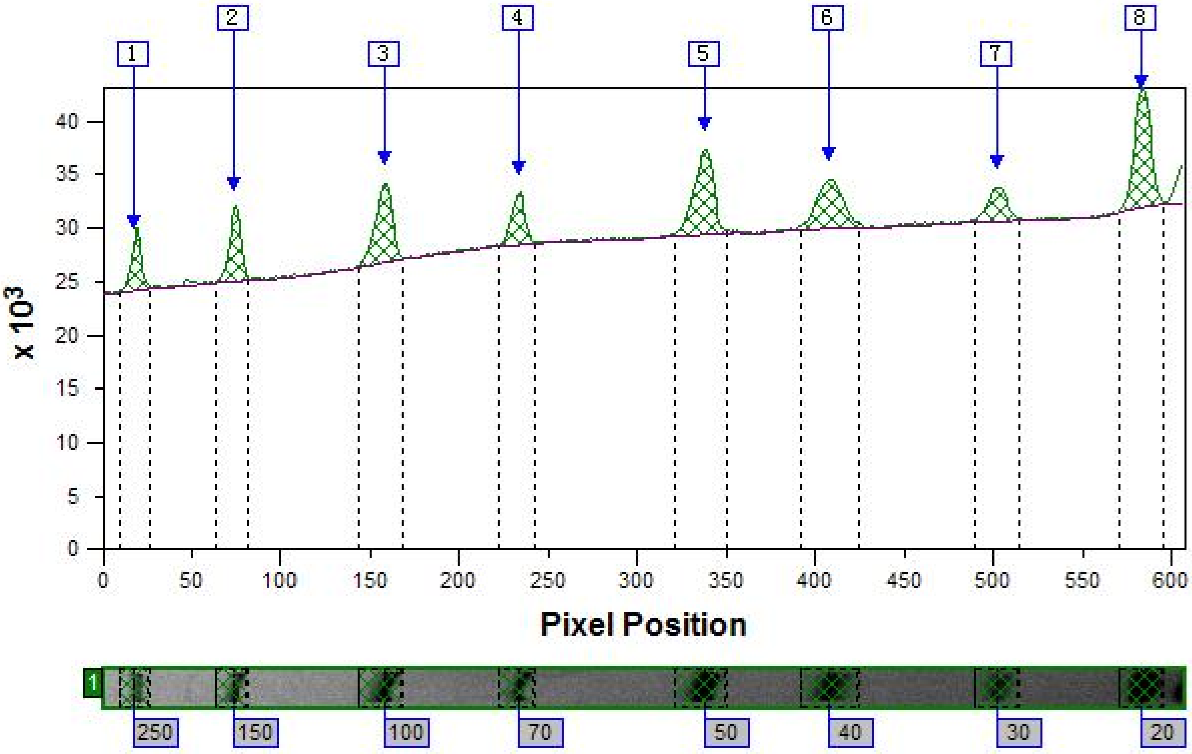

Band Table

Band No	Volume	Band %	MW
1	3284232.74	6.764	250.000
2	4529559.04	9.328	150.000
3	7155104.86	14.736	100.000
4	3739192.00	7.701	70.000
5	8868695.15	18.265	50.000
6	6877855.93	14.165	40.000
7	3558412.00	7.328	30.000
8	10543390.83	21.714	20.000

IQTL Report version 8.1
Time of Report: 9:03 2021-12-25 ( )

ImageQuant TL

Lane Data Report: 20211224-4 .tif, Lane 2

Summary

**Name:**	Lane 2

Background

**Background Type:**	Rolling Ball
**Rolling Ball Radius:**	200

Band Detection

**Automatic Detection:**	Yes
**Minimum Band Slope:**	50
**Median Filter:**	10
**Percentage Maximum Peak:**	0
**Maximum Peak Measure:**	Gel
**Edge Detection Method:**	Automatic Edges
**Band Positions Edited:**	No

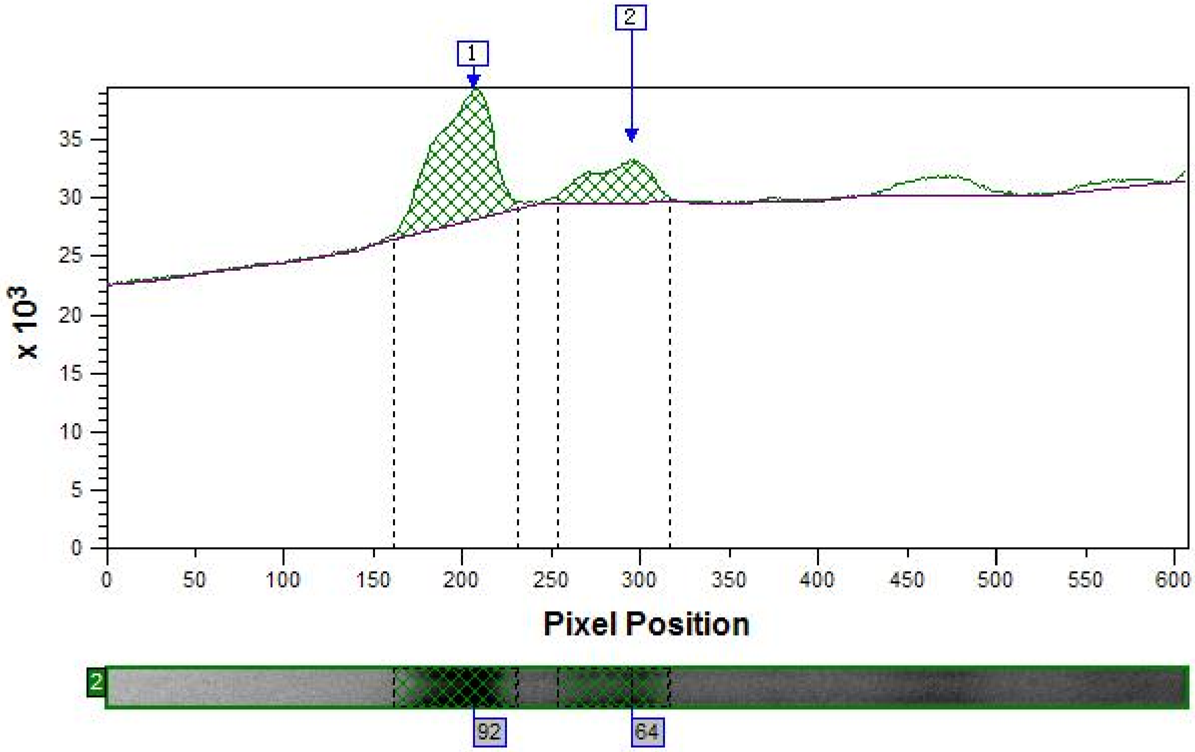

Band Table

Band No	Volume	Band %	MW
1	37891621.92	74.235	91.995
2	13150840.11	25.765	63.816

ImageQuant TL

Lane Data Report: 20211224-4   .tif, Lane 3

Summary

**Name:**	Lane 3

Background

**Background Type:**	Rolling Ball
**Rolling Ball Radius:**	200

Band Detection

**Automatic Detection:**	Yes
**Minimum Band Slope:**	50
**Median Filter:**	10
**Percentage Maximum Peak:**	0
**Maximum Peak Measure:**	Gel
**Edge Detection Method:**	Automatic Edges
**Band Positions Edited:**	No

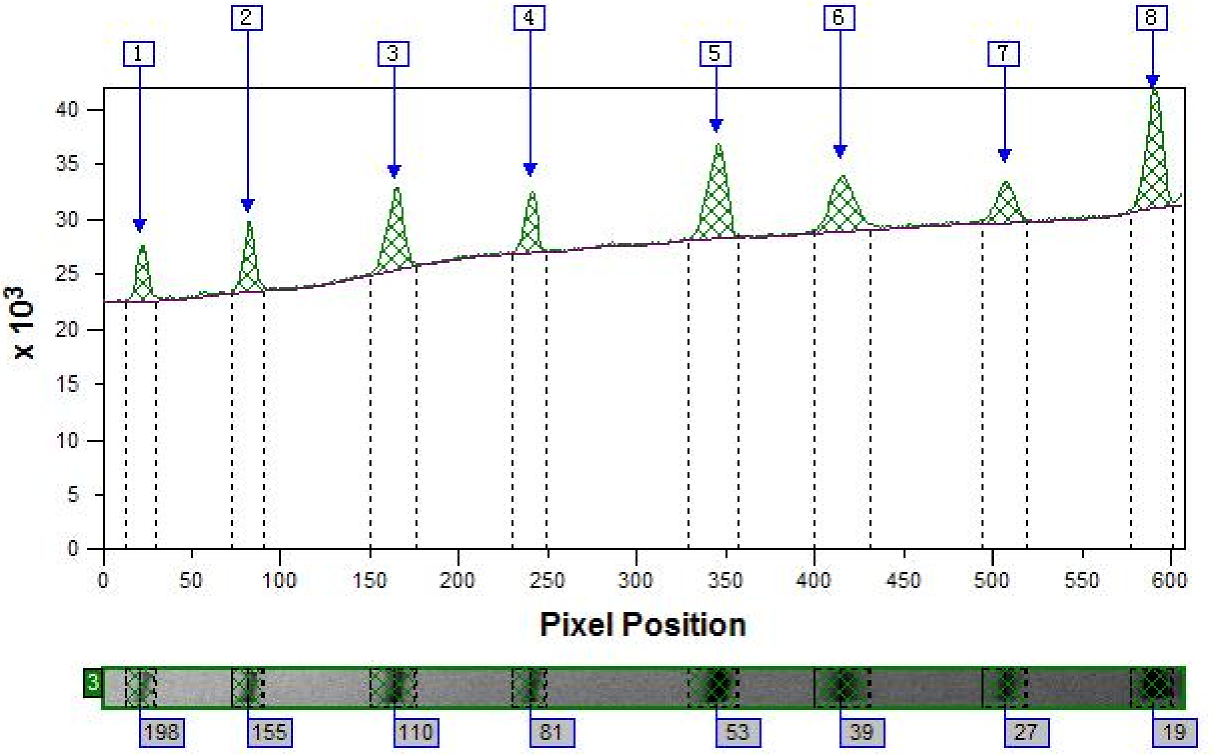

Band Table

Band No	Volume	Band %	MW
1	3271629.16	6.741	197.654
2	4231721.26	8.719	154.571
3	6991976.00	14.406	110.458
4	3760080.79	7.747	80.568
5	9103566.83	18.757	52.612
6	7137502.00	14.706	39.492
7	4069637.37	8.385	27.088
8	9968001.31	20.538	19.200

ImageQuant TL

Lane Data Report: 20211224-4   .tif, Lane 4

Summary

**Name:**	Lane 4

Background

**Background Type:**	Rolling Ball
**Rolling Ball Radius:**	200

Band Detection

**Automatic Detection:**	Yes
**Minimum Band Slope:**	50
**Median Filter:**	10
**Percentage Maximum Peak:**	0
**Maximum Peak Measure:**	Gel
**Edge Detection Method:**	Automatic Edges
**Band Positions Edited:**	No

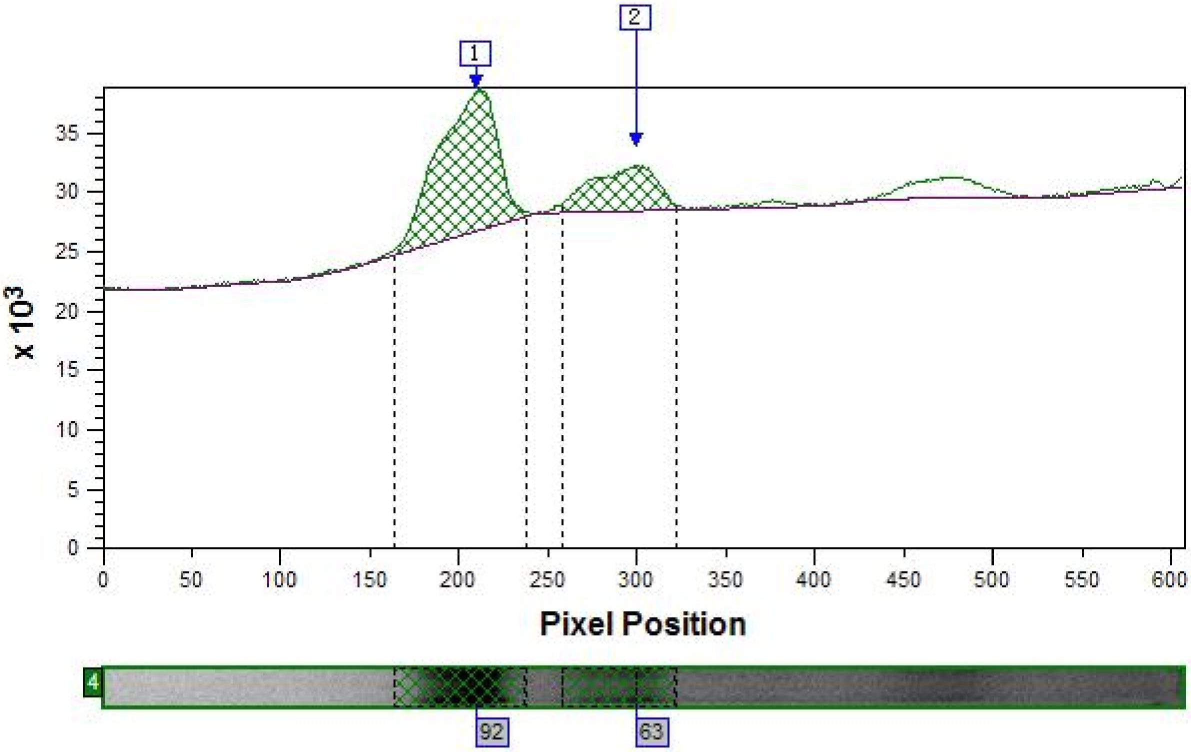

Band Table

Band No	Volume	Band %	MW
1	41222313.97	74.181	91.651
2	14347646.37	25.819	63.431

IQTL Report version 8.1
Time of Report: 9:03 2021-12-25 ( )

ImageQuant TL

Lane Data Report: 20211224-4 .tif, Lane 5

Summary

**Name:**	Lane 5

Background

**Background Type:**	Rolling Ball
**Rolling Ball Radius:**	200

Band Detection

**Automatic Detection:**	Yes
**Minimum Band Slope:**	50
**Median Filter:**	10
**Percentage Maximum Peak:**	0
**Maximum Peak Measure:**	Gel
**Edge Detection Method:**	Automatic Edges
**Band Positions Edited:**	No

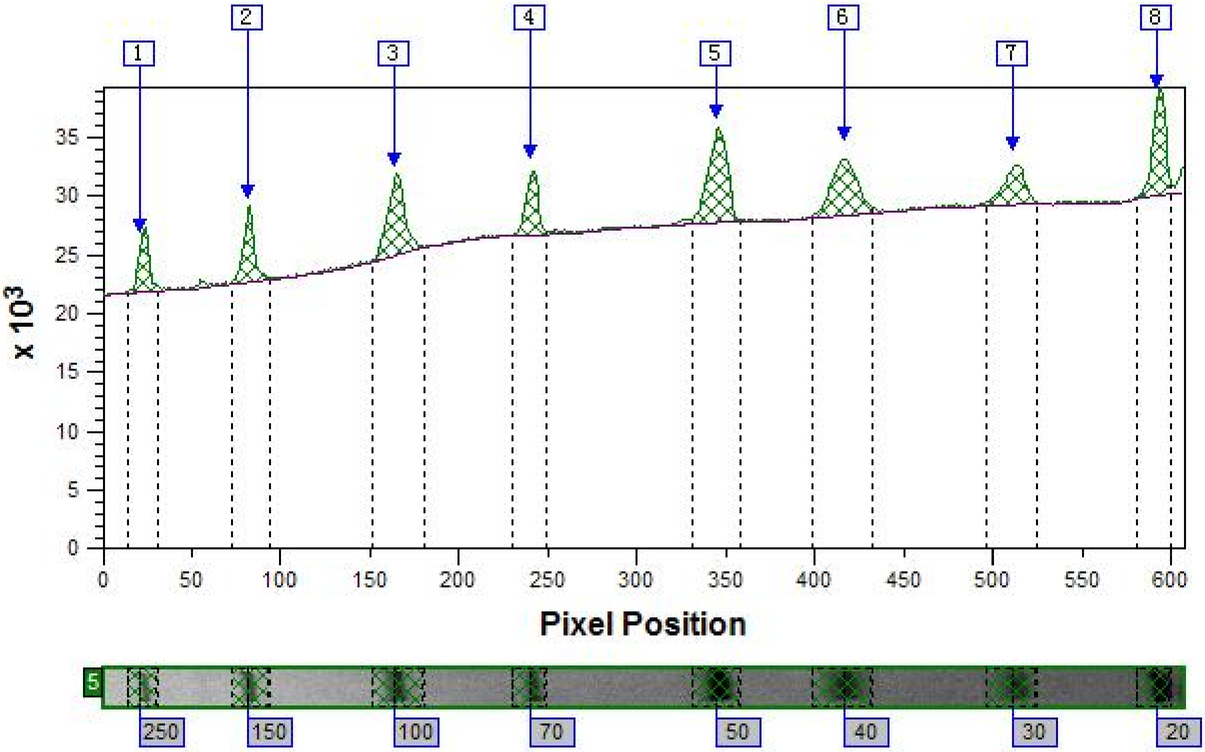

Band Table

Band No	Volume	Band %	MW
1	3037533.07	6.879	250.000
2	3995511.59	9.048	150.000
3	6787431.32	15.371	100.000
4	3760815.61	8.517	70.000
5	8808577.33	19.948	50.000
6	7249845.25	16.418	40.000
7	4136339.15	9.367	30.000
8	6381911.10	14.452	20.000

IQTL Report version 8.1
Time of Report: 9:03 2021-12-25 ( )

ImageQuant TL

Lane Data Report: 20211224-4 .tif, Lane 6

Summary

**Name:**	Lane 6

Background

**Background Type:**	Rolling Ball
**Rolling Ball Radius:**	200

Band Detection

**Automatic Detection:**	Yes
**Minimum Band Slope:**	50
**Median Filter:**	10
**Percentage Maximum Peak:**	0
**Maximum Peak Measure:**	Gel
**Edge Detection Method:**	Automatic Edges
**Band Positions Edited:**	No

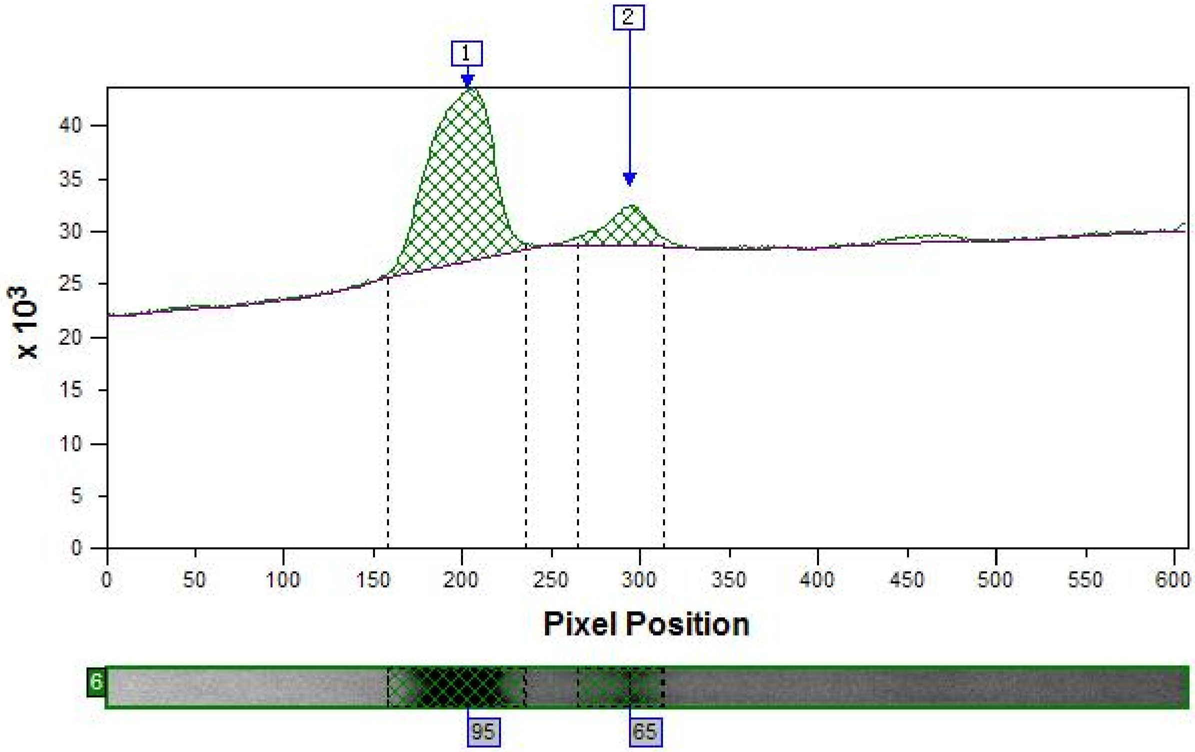

Band Table

Band No	Volume	Band %	MW
1	60352765.23	86.521	94.898
2	9402650.00	13.479	65.439
