# Supplementary figures and images for: A mosaic-type trimeric RBD-based COVID-19 vaccine candidate induces potent neutralization against Omicron and other SARS-CoV-2 variants
Source: eLife. 2022 Aug 25;11:e78633. doi: 10.7554/eLife.78633 (PMC9481243; doi:10.7554/eLife.78633)

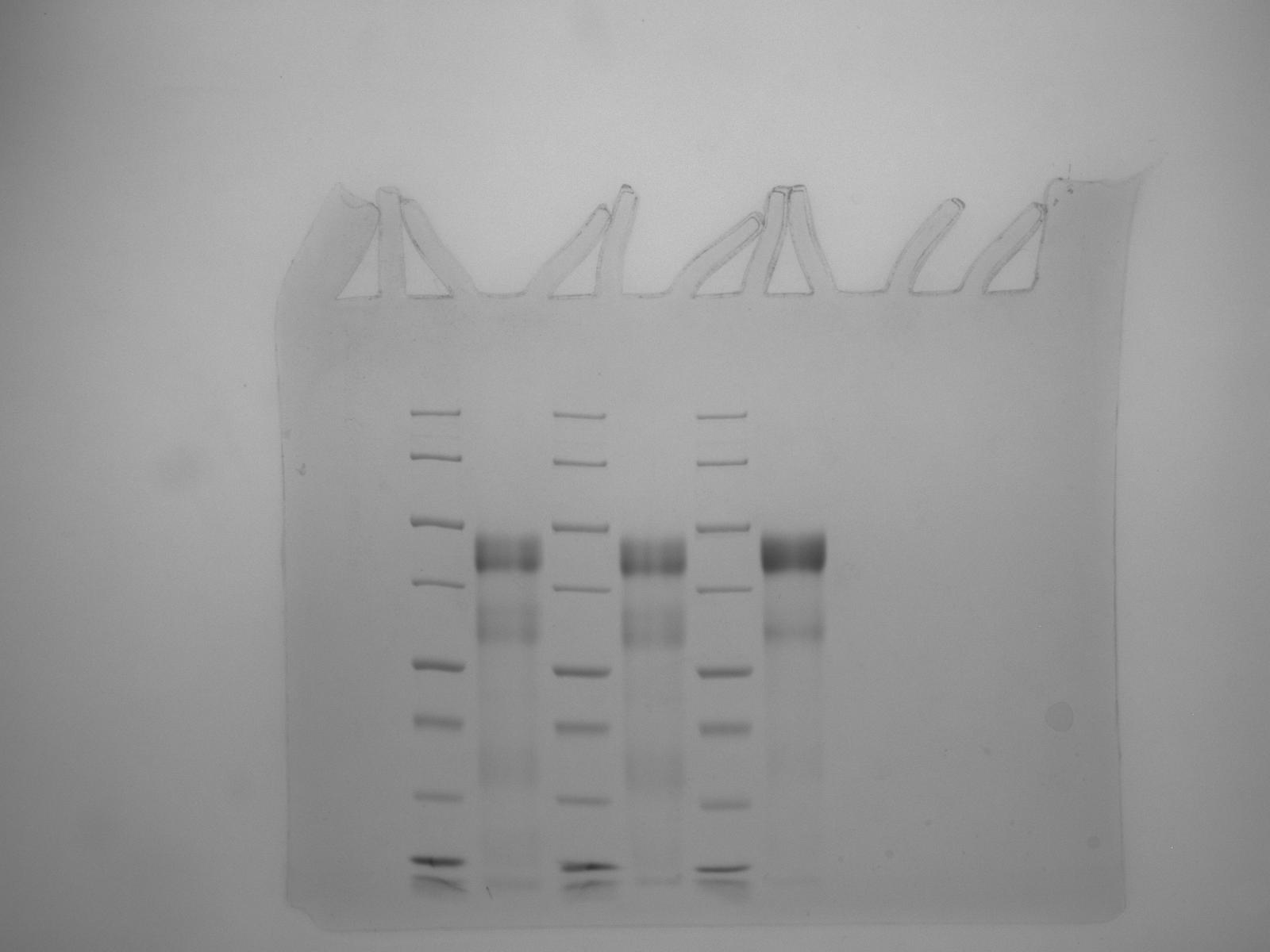

Supplement: Figure 1—source data 1. [file elife-78633-fig1-data1.zip › Figure 1 - source data 1/Figure 1B -original file of the full raw unedited SDS-PAGE gel.JPG]

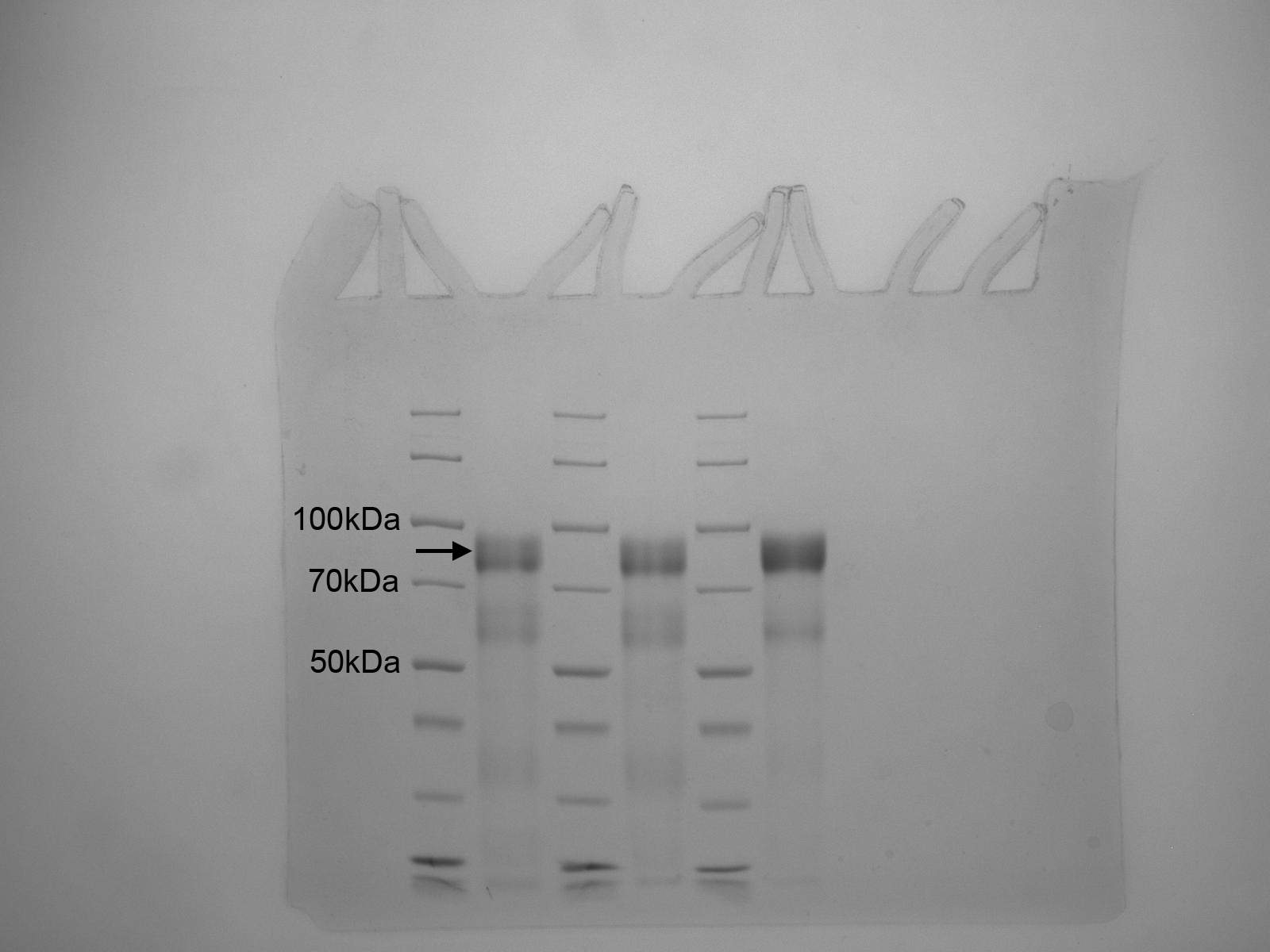

Supplement: Figure 1—source data 1. [file elife-78633-fig1-data1.zip › Figure 1 - source data 1/Figure 1B -the uncropped SDS-PAGE gel with the relevant band clearly labelled.jpg]
